# Shock Wave Lithotripsy Outcomes for Upper and Lower Ureteral Stones in Non-obese and Non-Pre-stented Adults: Is One Session Sufficient?

**DOI:** 10.7759/cureus.29592

**Published:** 2022-09-26

**Authors:** Jad K Alsmadi

**Affiliations:** 1 Department of General Surgery and Anesthesia, The Hashemite University, Faculty of Medicine, Zarqa, JOR

**Keywords:** lower ureter, upper ureter, ureteral stones, swl, shock wave lithotripsy

## Abstract

Purpose

This study aims to evaluate differences in shock wave lithotripsy (SWL) outcomes between upper and lower ureteral stones and identify patients who are likely to have a stone-free status after one session of SWL.

Materials and methods

After approval from the institutional review board and from a prospectively maintained database of 628 patients, 182 were retrospectively identified, who have had SWL for a single lower or upper ureteral stone and met the inclusion criteria. Age, body mass index (BMI), and stone size were similar among the groups. This study included non-pre-stented patients with solitary lower or upper ureteral radiopaque stones identified on non-contrast-enhanced computed tomography (NCCT), did not have acute obstruction, and had either normal body mass index (BMI) or overweight status. Patients were treated with Sonolith i-sys electroconductive lithotripter (focal length: 21 cm) (EDAP TMS, Vaulx‐en‐Velin, France). Success was defined as the absence of residual of any size or a residual of ≤2 mm on NCCT after one month, whereas failure was having fragments >2 mm or requiring surgical intervention. Post-SWL assessments were completed one week after every session with an X-ray of the kidney-ureter-bladder (KUB-XR) and NCCT after one month.

Results

The upper and lower ureteral stone-free rates (SFRs) were 95% and 64.7%, respectively. Of them, 65% and 45%, respectively, were stone-free after one session. The cohort having a stone-free status after one SWL session was similar in age, sex, BMI, and stone density. The upper ureteral stone arm has a significant chance for one SFR session with a larger stone size, shocks per session, and maximum power delivered. In the receiver operating characteristic (ROC) curves, the optimal cut point number of sessions of 1.5, mean stone density (MSD) of 895, and stone size of 10.5 mm are the most likely to have successful SWL in the ureter.

Conclusion

For patients having ureteral stones with favorable factors, SWL modality is effective and safe. Moreover, SWL can be done for one or two sessions only with the presence of favorable factors.

## Introduction

Over the last year, the coronavirus disease 2019 (COVID-19) pandemic has made treatment challenging in all hospitals worldwide, especially in terms of providing sufficient healthcare for inpatients and for non-emergent operations. This has brought back the importance of outpatient management, with no need for general anesthesia such as shock wave lithotripsy (SWL) for stones. The SWL procedure is advantageous in settings such as sedation and decreased emergency department (ED) visits after the treatment [1]. On the contrary, SWL has lower rates of stone clearance per visit, which is dependent on patient and stone factors and prevents its uniform use [2]. SWL for ureteral stones, either the upper or lower ureter, is a first choice therapy or at least a second choice, as reported in most urolithiasis guidelines [3].

Some research efforts have been made to minimize SWL failure by identifying unfavorable outcome predictors. Of these parameters, location in the lower ureter, size > 1 cm, obesity, obstructing stone, and lower stone heterogeneity index are associated with SWL failure [4,5]. Other predictors, such as age and skin-to-stone distance (SSD), have a remarkable effect on renal stones but are less remarkable in ureteral stones [6].

Worldwide, urolithiasis disease, with related disabilities and deaths, is increasing in incidence [7]. As part of urolithiasis, the prevalence of ureteral stones is not well known, and it can reach more than 15% of urolithiasis patients in acute settings [8].

Identifying patients who are most likely to get rid of their ureteral stones with less invasive procedures, such as SWL, will ultimately decrease the need for post-ureteroscopy (URS) procedure unplanned ED visits, auxiliary procedures, and additional costs.

In this series, wherein we focus on comparing upper and lower ureteral stones managed with SWL without previous treatment, some of the factors that negatively affect SWL outcomes had been avoided. This research was conducted to understand patients and stone characteristics that can identify patients who are likely to get rid of their stones from SWL in general and after one session only.

## Materials and methods

Study design

After approval from The Hashemite University Institutional Review Board (HU-IRB) (number 10/13/2020/2021), consecutive patients who had undergone SWL for solitary, radio-opaque, upper or lower ureteral calculi were identified on non-contrast-enhanced computed tomography (NCCT) and kidney-ureter-bladder X-ray (KUB-XR) and were retrospectively selected. The lower ureter was the portion below the upper border of the sacroiliac joint, and the upper ureter was the portion above the upper border of the sacroiliac joint. Patients underwent SWL between January 2021 and February 2022, and they were included if they were adults (18 years old or more), had body mass index (BMI) between 18.5 and <30 kg/m^2^, and had had an NCCT before treatment and if SWL had been performed for a single ureteral stone. The exclusion criteria were as follows: underweight patients (BMI ≤ 18.5 kg/m^2^), obese patients (BMI ≥ 30 kg/m^2^), patients with acute obstruction or moderate-severe hydronephrosis on NCCT or ureteral stents, and patients who had been surgically treated for the same stone before. All patients signed an informed consent agreeing to supply their anonymous information for research purposes.

All patients with a stone size of <1 cm had been treated with and failed observation or conservative management according to the American Urological Association (AUA) guidelines [9]. Moreover, none of the patients had an acute obstruction or severe hydroureteronephrosis (HUN) on NCCT, as these subsets of patients will have undergone ureteroscopy (URS) as a routine procedure in our center.

SWL treatment

Patients were treated using the Sonolith i-sys electroconductive lithotripter (EDAP TMS, Vaulx‐en‐Velin, France), with a maximum focal depth of 21 cm. The procedure was routinely performed with a pre-check for vital signs by a nurse. Then, positioning was performed by a specialized radiology technician; for the upper ureter, the patient was asked to lie down on his back with either head-up or head-down, depending on the side of the stone, and for the lower ureter, the patient was asked to lie down on his belly. Analgesia was routinely administered as injectable intramuscular nonsteroidal anti-inflammatory drugs and sedation if needed. Stone localization was performed under fluoroscopy guidance combined with ultrasonography (Flex Focus 200, BK Medical, Herlev, Denmark) for ultrasonically visible ureteral stones, wherein the ultrasound probe was connected to the machine with Visio-Track technology for continuous real-time tracking and treatment monitoring of the stone. The shock wave frequency was routinely set to 2 Hz. Power was started at 5% (about 70 Joules) for the first 100 shocks and then increased every 50 shocks in 5% increments to 80% or to the maximum power tolerated by the patient. Treatment was terminated when 1,000 Joule energy was reached (the maximum is 1,400 Joule), fragmentation of the stone was achieved, or the patient can no longer tolerate the procedure.

The patients underwent a plain X-ray of the kidney-ureter-bladder (KUB) one week following each treatment session to assess the response. NCCT was routinely scheduled for patients after one month of having either a non-visualized stone on SWL fluoroscopy or after three sessions with no or partial response. Overall treatment success was defined as the absence of stones on one-month NCCT or the presence of clinically insignificant residual fragments of size ≤ 2 mm. SWL failure was defined as the persistence of fragments of size > 2 mm at one month, inability to tolerate SWL, or the need for surgical intervention.

Statistical analyses

The baseline demographic and clinical characteristics of patients undergoing SWL in the upper and lower ureters were compared using the chi-square test for categorical variables and independent-samples t-test or Mann-Whitney U test for normally distributed and non-normally distributed continuous variables, respectively. Patients with successful SWL after one session were further analyzed using the same statistical method. Receiver operating characteristic (ROC) curve and area under the curve (AUC) for continuous characteristics were used to define treatment success cutoff points. Statistical significance was defined as p < 0.05. All data analyses were performed using Statistical Package for the Social Sciences (SPSS) version 24 (IBM Corp., Armonk, NY, USA).

## Results

Among 628 patients who underwent SWL, 182 met our inclusion criteria. Patient and stone information is summarized in Table 1. SWL treatment information is summarized in Table 2. The cohort did not show any significant difference between upper and lower ureteral stone treatments in terms of age, BMI, stone size, or the number of shocks per SWL session. The lower ureteral SWL treatment group had denser stones (836 versus 749, p = 0.06), needed more SWL sessions (1.8 versus 1.4, p = 0.001), and required lower maximal power (51.5% versus 63.2%, p < 0.0001). The success rates for upper and lower ureteral stones were 95% and 64.7%, respectively, with an overall success rate of 78%.

**Table 1:** Patient and Stone Characteristics SD: standard deviation, BMI: body mass index, MSD: mean stone density, HU: Hounsfield units, mm: millimeter

	Upper ureteral stone	Lower ureteral stone	P value
Number of patients	80	102	
Age in years (mean ± SD)	43.1 ± 10.5	44.8 ± 14.3	0.41
Gender: male/female	28/52	16/86	0.003
BMI (mean ± SD)	26.6 ± 2.7	25.9 ± 2.4	0.08
Stone laterality: right/left	32/48	86/16	<0.0001
MSD (HU mean ± SD)	748.5 ± 232.7	835.69 ± 303.73	0.06
Stone size in mm (mean ± SD)	9.5 ± 2.9	9.06 ± 3.4	0.14

**Table 2:** SWL Treatment Characteristics SWL: shock wave lithotripsy, SFR: stone-free rate, SD: standard deviation

	Upper ureteral stone	Lower ureteral stone	P value
Number of patients	80	102	
Number of sessions (mean ± SD)	1.4 ± 0.7	1.8 ± 0.9	0.001
Number of waves/session (mean ± SD)	3,637.5 ± 318	3,670.6 ± 462.1	0.78
Maximum power reached (mean ± SD)	63.2% ± 13%	51.5% ± 17%	<0.0001
SFR number (fraction)	76 (95%)	66 (64.7%)	<0.0001

Only 98 (53.8%) patients had a successful outcome after the first session of SWL (Table 3). Age and BMI were not significantly different between the two groups. Female and male patients were equally distributed between the upper and lower ureteral arms with regard to success after one session. Additionally, the mean stone density (MSD) showed no significant difference. Patients with lower ureteral stones who had a successful outcome after one session had a lower stone size (7.3 versus 8.5 mm, p = 0.002), a lower number of shock waves being delivered per session (3,552 versus 3,673, p = 0.04), and lower maximal power reached per session (48.1% versus 66.7%, p < 0.0001). Regarding the laterality of the stones, the cohort showed more right-sided lower ureteral stones and more left-sided upper ureteral stones, and within the cohort of patients with one successful session, it remained the same.

**Table 3:** Patients With One Session Stone-Free Outcome SD: standard deviation, BMI: body mass index, MSD: mean stone density, HU: Hounsfield units, mm: millimeter

	Upper ureteral stone	Lower ureteral stone	P value
Number of patients	52 (65%)	46 (45%)	0.007
Age in years (mean ± SD)	41.5 ± 10.3	40.3 ± 14.6	0.64
Gender: male/female	14/38	10/36	0.55
BMI (mean ± SD)	25.8 ± 2.8	24.8 ± 2.2	0.05
Stone laterality: right/left	16/36	36/10	<0.0001
MSD (HU mean ± SD)	736.2 ± 250	663.9 ± 182	0.26
Stone size in mm (mean ± SD)	8.5 ± 2.9	7.3 ± 2.8	0.002
Number of shocks/session (mean ± SD)	3,673 ± 278	3,552 ± 330	0.04
Maximum power reached (mean ± SD)	66.7% ± 112%	48.1% ± 16.6%	<0.0001

A comparison of ROC curves for assessing the effect of several parameters on being stone-free for ureteral stones is shown in Table 4. The optimal number of SWL sessions in the ureter was ≤1.5 sessions with a 73.2% stone clearance rate. An MSD of ≤895 will have 81.7%, and a stone average size of ≤10.5 mm will have a 76.1% success rate.

**Table 4:** ROC Curve Statistics ROC: receiver operating characteristic, AUC: area under the curve, TPR: true-positive rate, FPR: false-positive rate, SWL: shock wave lithotripsy, MSD: mean stone density, mm: millimeter, max: maximum Curves with an AUC of 0.7 or more were considered of excellent discrimination power, and stone size AUC was included.

	AUC	Confidence interval (95%)	Optimal cut point	Sensitivity TPR	1- Specificity FPR
Number of SWL sessions	0.942	0.893-0.991	1.5	73.2%	5%
MSD	0.808	0.722-0.894	895	81.7%	30%
Stone size	0.696	0.593-0.8	10.5 mm	76.1%	35%
Age	0.67	0.58-0.76	45 years	64.8%	35%
BMI	0.664	0.571-0.758	27.3 kg/m^2^	63.4%	35%
Number of waves/session	0.661	0.562-0.76	3,750	66.2%	35%
Max power reached	0.557	0.459-0.655	67.5%	71.8%	65%

## Discussion

This study showed that SWL is an effective approach in ureteral stone management with an overall success of 78% after selection, as the reported overall success rate for SWL in the ureter is 72% [9]. In selecting the appropriate candidates for successful SWL treatment, a high percentage of them will need one SWL session only or a second one at most. Conversely, continuing the treatment for a third or more sessions should be discouraged. Moreover, lower ureteral stones had lower SFRs than upper ureteral stones after SWL, which is consistent with the literature.

SWL is a first-line treatment for ureteral calculi side by side with URS [2,9]. The main downside of SWL is the lower SFR per procedure, but with comparable SFRs later [2]. On the contrary, SWL has the advantages of lower morbidity, lower complication rates, and shorter hospital stay [9].

Recently, the COVID-19 pandemic has raised the interest of doctors and patients to reduce the chance of acquiring infection inside hospitals during operations and to reduce the close exposure of anesthetists and medical personnel to patients during possible general anesthesia.

The success rate of SWL in the management of ureteral stones has been reported to be lower than that of URS in all parts of the ureter [9]. However, a recent systematic review and meta-analysis showed that the SFR for SWL, in the upper ureter, was lower than that for URS, over the first four weeks only; thereafter, SWL and URS had equal SFRs after a three-month follow-up [10]. For lower ureteral stones, SWL has a lower reported SFR than URS, but it is still the choice in patients refusing URS [9].

The outcome of SWL for ureteral stones is dependent on several patient- and stone-related factors such as obesity and BMI, size and density of the calculi, and degree of ureteral obstruction (HUN) [6,11,12]. In addition, the presence of stents may have a detrimental effect on outcomes after SWL for ureteric stones [13].

In our study, we took measures to reduce some influencing factors by selecting a non-obese population, with patients who had not been treated before and who had no severe HUN on NCCT. After this selection, the SFRs after SWL in the upper and lower ureteral stones were 95% and 65%, respectively.

Because SWL for ureteral stones can have a low success rate after a failed initial treatment, patients who had an SFR after one SWL session were compared separately [14]. Patients with SFR after one session constituted 54% of the total and were comparable in age, sex, BMI, and MSD. Patients with upper ureteral stones had larger stones, more waves delivered per session, and higher maximal power that could be reached per session. Moreover, in this study, the proportion of patients with upper ureteral stones who attained an SFR from one SWL session was 65%, while it was only 45% in those with lower ureteral stones.

The ROC curves (with an AUC of approximately 70% and above) of this cohort have depicted that the appropriate number of SWL sessions is one or two sessions, stone density of up to 895, and stone size of up to 1.05 cm.

This study has some limitations, such as the retrospective design and too many exclusions in selecting the targeted patients. Despite these, the study still represents a high fraction of SWL patients. Further prospective studies focusing on making categories for SWL treatment success prediction are warranted.

## Conclusions

For patients having ureteral stones with favorable factors for SWL, this modality is effective and safe. Moreover, SWL can be done for one or two sessions with high success rates. However, lower ureteral stones are still having lower SFRs than the upper ureteral stones after SWL. Favorable factors for SWL success after one or two sessions include normal weight for patients, lower size and density for the stone, and the presence of neither JJ stent nor obstruction due to the stone.
